# Cell-specific targeting of lentiviral vectors mediated by fusion proteins derived from Sindbis virus, vesicular stomatitis virus, or avian sarcoma/leukosis virus

**DOI:** 10.1186/1742-4690-7-3

**Published:** 2010-01-25

**Authors:** Xian-Yang Zhang, Robert H Kutner, Agnieszka Bialkowska, Michael P Marino, William B Klimstra, Jakob Reiser

**Affiliations:** 1 Gene Therapy Program, Department of Medicine, Louisiana State University Health Sciences Center, New Orleans, Louisiana 70112, USA; 2 Leonard M Miller School of Medicine, University of Miami, Miami, Florida 33136, USA; 3 Emory University School of Medicine, Atlanta, Georgia 30322, USA; 4 Center for Biologics Evaluation and Research, US Food and Drug Administration, Bethesda, Maryland 20892, USA; 5 Center for Vaccine Research, University of Pittsburgh, Pittsburgh, Pennsylvania 15261, USA

## Abstract

**Background:**

The ability to efficiently and selectively target gene delivery vectors to specific cell types *in vitro *and *in vivo *remains one of the formidable challenges in gene therapy. We pursued two different strategies to target lentiviral vector delivery to specific cell types. In one of the strategies, vector particles bearing a membrane-bound stem cell factor sequence plus a separate fusion protein based either on Sindbis virus strain TR339 glycoproteins or the vesicular stomatitis virus G glycoprotein were used to selectively transduce cells expressing the corresponding stem cell factor receptor (c-kit). An alternative approach involved soluble avian sarcoma/leukosis virus receptors fused to cell-specific ligands including stem cell factor and erythropoietin for targeting lentiviral vectors pseudotyped with avian sarcoma/leukosis virus envelope proteins to cells that express the corresponding receptors.

**Results:**

The titers of unconcentrated vector particles bearing Sindbis virus strain TR339 or vesicular stomatitis virus G fusion proteins plus stem cell factor in the context of c-kit expressing cells were up to 3.2 × 10^5 ^transducing units per ml while vector particles lacking the stem cell factor ligand displayed titers that were approximately 80 fold lower. On cells that lacked the c-kit receptor, the titers of stem cell factor-containing vectors were approximately 40 times lower compared to c-kit-expressing cells.

Lentiviral vectors pseudotyped with avian sarcoma/leukosis virus subgroup A or B envelope proteins and bearing bi-functional bridge proteins encoding erythropoietin or stem cell factor fused to the soluble extracellular domains of the avian sarcoma/leukosis virus subgroup A or B receptors resulted in efficient transduction of erythropoietin receptor or c-kit-expressing cells. Transduction of erythropoietin receptor-expressing cells mediated by bi-functional bridge proteins was found to be dependent on the dose, the correct subgroup-specific virus receptor and the correct envelope protein. Furthermore, transduction was completely abolished in the presence of anti-erythropoietin antibody.

**Conclusions:**

Our results indicate that the avian sarcoma/leukosis virus bridge strategy provides a reliable approach for cell-specific lentiviral vector targeting. The background levels were lower compared to alternative strategies involving Sindbis virus strain TR339 or vesicular stomatitis virus fusion proteins.

## Background

Targeted vector delivery has been approached in a number of ways [1]. For example, the host range of retroviral vectors including that of lentiviral vectors can be expanded or altered by a process known as pseudotyping. Pseudotyped retroviral vectors consist of vector particles bearing envelope (Env) glycoproteins derived from other enveloped viruses. Such particles possess the tropism of the virus from which the glycoprotein was originally derived [2].

It has been challenging to develop lentiviral vectors that display a reduced tropism for the natural receptor and an increased specificity for a chosen receptor to allow targeted transduction of specific cell types *in vitro *and *in vivo *[3]. Such targeting approaches have involved engineered versions of the Sindbis virus E2 glycoprotein bearing either a *Staphylococcus aureus *protein A domain [4-14] or single chain antibody fragments fused in-frame to the E2 glycoprotein coding region [15], allowing antibody-mediated cell targeting in the presence of the Sindbis virus E1 fusion protein. A related strategy that uncouples the target cell recognition function from the fusion function presents them as separate proteins on the vector's surface. This has proven more flexible and has facilitated cell-specific targeting of gammaretroviral [16] and lentiviral vectors [17-22]. One drawback of these approaches is that background transduction levels are substantial even in the absence of the ligand or when using cells lacking the corresponding receptors due to the leakiness of the mutations that were introduced into the Sindbis virus E2 glycoprotein for abolishing cell binding.

Alternative approaches for cell-specific targeting of alpharetroviral and gammaretroviral vectors have been described. These involve the use of ligand proteins or cell-specific antibodies as a bridge to target vectors carrying unmodified avian sarcoma/leukosis virus (ALV) Env proteins to specific cells *in vitro *[23-27]. This system is attractive because of its flexibility to accommodate cell-specific ligands without impacting the Env glycoprotein. Also, the reported background transduction levels were low.

Here we show that HIV-1-based lentiviral vectors are able to form efficient pseudotypes with Env glycoproteins derived from ALV subgroups A and B. Furthermore, vectors pre-incubated with bi-functional bridge proteins encoding human erythropoietin (Epo) or stem cell factor (SCF) fused to the soluble extra-cellular domains of the ALV subgroup A and B receptors resulted in efficient transduction of mammalian cells expressing the human erythropoietin receptor (EpoR) or c-kit. We also show that targeted cell transduction can be achieved using lentiviral vectors particles bearing a membrane-bound form of SCF in conjunction with an independent fusion domain derived from VSV-G [28,29] or the glycoproteins derived from a non-heparan sulfate-binding strain of Sindbis virus [30].

## Results

### Targeting of c-kit-expressing cells with lentiviral vectors bearing Sindbis virus strain TR339 glycoproteins and human SCF

**Figure 1:**
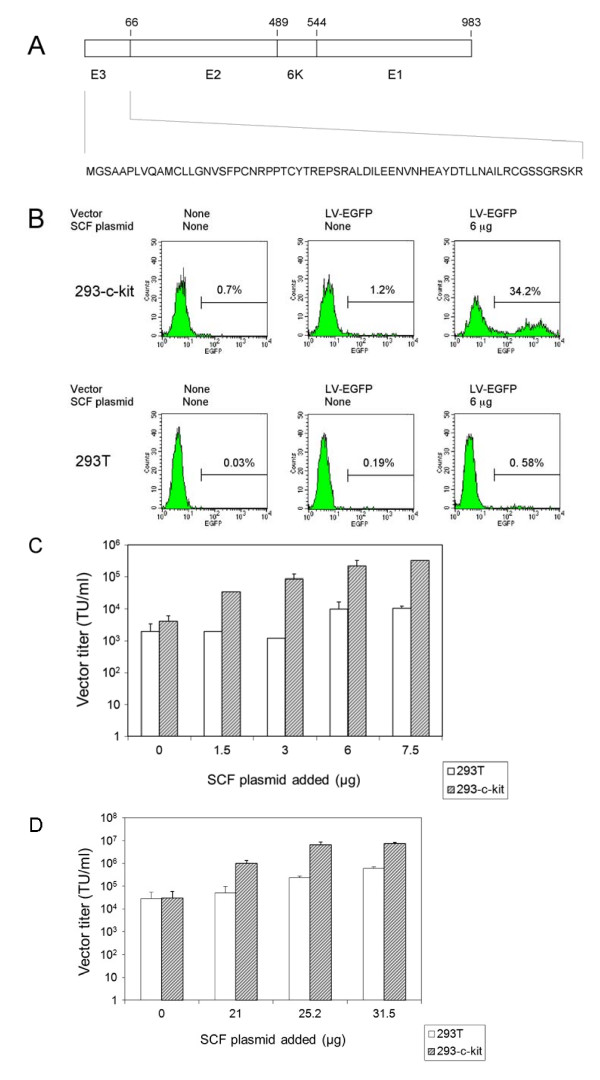
**Transduction of c-kit-expressing 293 cells using lentiviral vectors pseudotyped with the Sindbis virus strain TR339 glycoproteins and bearing SCF**. (A) Schematic representation of the modified Sindbis virus strain TR339 proteins. The sequence of the modified E3 protein is shown. It encodes 66 amino acids including the signal peptide for the E2 protein. E2 consists of 423 amino acids, and the 6K and E1 proteins encode 55 and 439 amino acids, respectively. The numbers refer to the ends of the respective protein domains. (B) Representative FACS profiles. Top panels: 293-c-kit cells transduced with vectors prepared using 0 and 6 μg of the SCF-encoding pUB-HuMGF plasmid; Top left panel: Untransduced cells. Bottom panels: 293T cells transduced with vectors prepared using 0 and 6 μg of the SCF-encoding pUB-HuMGF plasmid; Bottom left panel: Untransduced cells. The percentages of EGFP-positive cells are indicated. (C) Unconcentrated vector titers on 293 cells expressing c-kit (striped bars) and on 293T cells (open bars) three days after transduction. The titers shown represent the mean ± standard deviation (SD) obtained from three independent experiments. (D) Determination of concentrated vector titers. Vectors were concentrated 300 fold by ultracentrifugation. The titers shown represent the mean ± SD from three to six independent experiments. Striped bars: 293 cells expressing c-kit; Open bars: 293T cells.

We first tested a cell-targeting approach using an EGFP-expressing lentiviral vector (LV-EGFP) pseudotyped with modified glycoproteins derived from the Sindbis virus TR339 strain [30] and bearing a membrane-bound version of SCF. Such membrane-bound forms of SCF have been shown before to be biologically active and to facilitate targeted retroviral transduction [16,31]. Unlike cell culture-adapted strains of Sindbis virus that exhibit efficient initial attachment to cell surface heparan sulfate receptors, the TR339 strain exhibited little to no interaction with heparan sulfate and low binding to cell surfaces while it was still able to chaperone the E1 fusion protein [32]. Based on these findings, we surmised that the Sindbis virus strain TR339 E2 and the E1 glycoproteins would promote vector uptake by target cells containing a specific receptor (such as c-kit) provided that an appropriate ligand is also present on the vector particle. An outline of the TR339 proteins used in this work is presented in Fig. 1A. A Western blot analysis of vector particles that had been concentrated by ultracentrifugation was carried out to test whether SCF was stably associated with such particles. Consistent with earlier results [16,31], SCF was found to be present in concentrated lentiviral vector preparations (data not shown). Such vector particles were then tested functionally. Fig. 1B shows that lentiviral vector particles pseudotyped with strain TR339-derived glycoproteins (SB-Env) and bearing SCF resulted in efficient transduction of 293-c-kit cells (Fig. 1B, top panels and Fig. 1C and 1D) while vector particles lacking SCF displayed lower transduction efficiencies (Fig. 1B top panels). The titers of unconcentrated SB-Env pseudotypes bearing SCF on c-kit-expressing 293 cells were approximately 80 fold higher than those observed with vectors lacking SCF (3.2 ± 0.04 × 10^5 ^transducing units, TU/ml vs. 4.1 ± 2.0 × 10^3 ^TU/ml). The titers on 293T cells that do not express c-kit were 2.0 ± 1.4 × 10^3 ^TU/ml for vector particles lacking SCF and 1.1 ± 0.1 × 10^4 ^TU/ml for particles containing SCF (Fig. 1B, bottom panels and Fig. 1C and 1D). This result indicates that a majority of the transduction events were mediated by SCF-c-kit interactions. Fig. 1D shows that vector particles bearing SB-Env plus SCF retained the ability to transduce 293-c-kit cells after concentration by ultracentrifugation. The titers on 293-c-kit cells were 3.1 ± 1.3 × 10^4 ^TU/ml before concentration and 6.5 ± 2.1 × 10^6 ^TU/ml after a 300 fold concentration.

On MO7-e cells, a cell line expressing c-kit [31,33] lentiviral vectors pseudotyped with SB-Env and bearing SCF revealed titers around 1.2 ± 0.4 × 10^4 ^TU/ml while vectors lacking SCF revealed titers < 10^2 ^TU/ml (data not shown). These results confirm the results obtained with the 293-c-kit cells.

### Cell specific targeting using lentiviral particles displaying human SCF and a fusion domain derived from VSV-G

**Figure 2:**
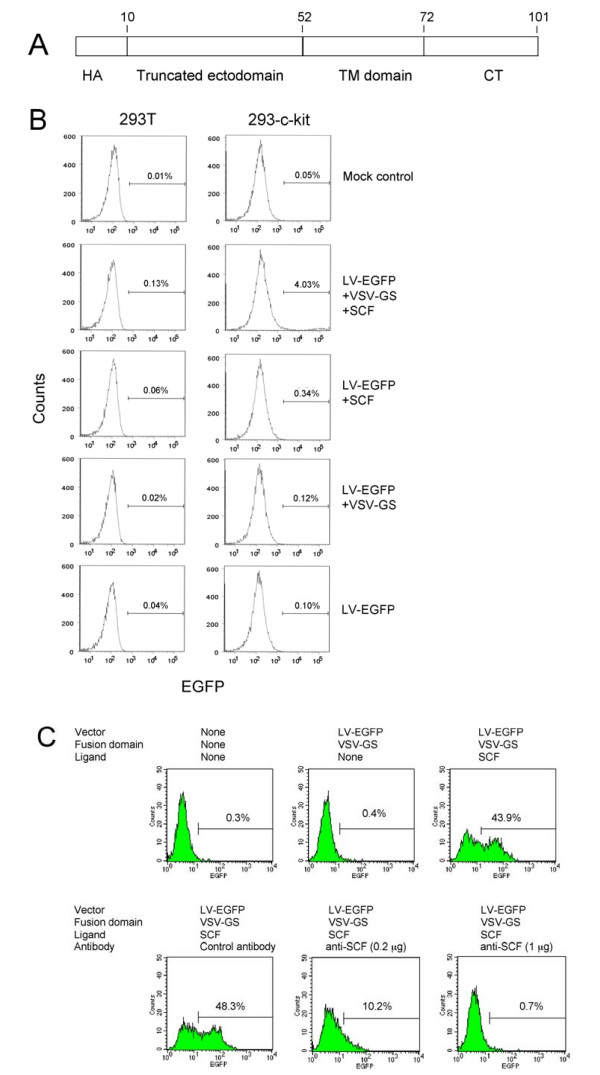
**Transduction of c-kit-expressing cells using lentiviral vectors bearing a VSV-G-derived fusion domain and SCF**. (A) Schematic representation of the VSV-GS fusion protein. HA: An HA epitope sequence (KYPYDVPDYA) was included to facilitate detection of the VSV-GS protein. The truncated ectodomain, the transmembrane domain (TM) and the cytoplasmic tail (CT) are indicated. The numbers refer to the ends of the respective protein domains. (B) Transduction of 293T and 293-c-kit cells using LV-EGFP vector particles bearing the VSV-GS fusion domain and/or SCF. Additional controls included LV-EGFP vector particles bearing VSV-GS but lacking SCF and vector particles lacking both VSV-GS and SCF. Left panels: 293T cells; Right panels: 293-c-kit cells. Cells were analyzed by FACS three days later. FACS profiles of representative assays are shown. (C) Transduction of MO7-e cells using EGFP-encoding lentiviral vector particles displaying SCF plus the VSV-GS fusion domain. Cells were transduced by spinoculation. Vector titers were determined by FACS analysis three days later. Goat anti-hSCF was added during transduction of the samples shown in the lower center and lower right panels. Normal goat serum referred to as control serum was added to the sample shown in the bottom left panel.

We next investigated an alternative lentiviral vector targeting strategy in which a membrane-bound form of SCF was incorporated into EGFP-encoding lentiviral (LV-EGFP) particles containing a truncated version of the VSV-G glycoprotein (VSV-GS). VSV-GS consists of a truncated ectodomain bearing a membrane-proximal stem region and the native trans-membrane and cytoplasmic domains (Fig. 2A). The VSV-GS protein was previously shown to enhance the cell-cell fusion activity of heterologous Env proteins [28,29]. LV-EGFP particles bearing VSV-GS resulted in specific transduction of 293 cells expressing c-kit as judged by FACS (Fig. 2B). It was interesting to note that vector particles bearing SCF but no fusion domain resulted in low but detectable transduction of 293-c-kit cells. Low but reproducible transduction of 293-c-kit cells with vectors containing SCF but lacking VSV-GS was also observed with β-galactosidase-encoding vectors as judged by X-Gal staining of transduced cells (data not shown).

As shown in Fig. 2C, LV-EGFP particles decorated with SCF and containing VSV-GS resulted in the appearance of EGFP-positive MO7-e cells (upper right panel) while LV-EGFP particles lacking SCF did not (upper middle panel). Furthermore, transduction was abolished following addition of a polyclonal goat anti-SCF antibody (Fig. 2C, lower center and lower right panels) but not after the addition of normal goat serum. The vector titers using MO7-e cells expressing c-kit were 6.2 ± 1.5 × 10^4 ^TU/ml. Following a 250-fold concentration by ultracentrifugation, the titers reached 6.9 ± 3.8 × 10^6 ^TU/ml (data not shown). This shows that the ability of VSV-GS-containing vector particles to transduce cells was only minimally affected by ultracentrifugation.

### Transduction of human cells expressing a chimeric ALV receptor using lentiviral vectors pseudotyped with the ALV-A or ALV-B Env glycoproteins

**Figure 3:**
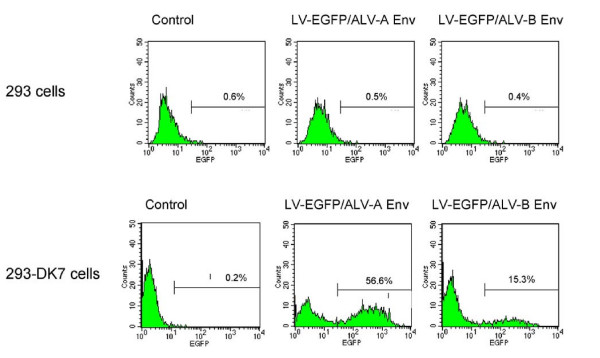
**Transduction of 293 cells expressing a chimeric TVA/TVB receptor using lentiviral vectors pseudotyped with the ALV-A and ALV-B Env glycoproteins**. Upper panel: 293 cells transduced with LV-EGFP vectors pseudotyped using ALV-A Env, or ALV-B Env. Lower panel: 293 DK-7 cells transduced using LV-EGFP pseudotyped with ALV-A Env or ALV-B Env. 1.64 × 10^3 ^pg of p24 were used for ALV-A Env pseudotypes, and 0.7 × 10^3 ^pg of p24 for ALV-B pseudotypes. Control refers to mock-transduced cells. FACS profiles of representative assays are shown.

We next wished to determine the potential of a bridge targeting strategy for cell-specific lentivirus vector targeting involving cell-specific ligands linked in-frame to the soluble extracellular domains of ALV receptors referred to as TVA and TVB, respectively. Such bridge strategies were used before to target alpharetroviral and gamaretroviral vectors carrying ALV Env proteins to mammalian cells bearing specific receptors [23-27]. To evaluate this approach, lentiviral vector pseudotypes prepared using glycoproteins derived from ALV subgroups A or B (referred to as ALV-A Env and ALV-B Env, respectively) were first tested on 293 cells expressing the corresponding TVA and TVB receptors. For transduction, vector particle numbers were adjusted based on p24 antigen levels. As shown in Fig. 3 (lower panels) and Table 1, 293-DK7 cells expressing a chimeric TVA-TVB receptor [34] were transduced with relatively high efficiency reaching titers between 10^5 ^to 10^6 ^TU/ml (Table 1) while such pseudotypes did not transduce 293 cells lacking the corresponding receptors. Following a 250-fold concentration by ultracentrifugation, titers between 10^7 ^to 10^8 ^TU/ml were obtained (Table 1). This indicated that such pseudotypes survived concentration by ultracentrifugation.

**Table 1:** Comparison of titers of lentiviral vectors pseudotyped with ALV-A or ALV-B Env glycoproteins in cell lines expressing TVA and TVB receptors, EpoR or c-kit

Vector pseudotypes	Cells	Titers (TU/ml)^d^
ALV-A	293 DK-7^a^	1.57 ± 0.47 × 10^8^
ALV-A/Epo	293 or 293T 293T-EpoR^b^	<10^4^ 8.60 ± 0.70 × 10^7^
ALV-A/SCF	MO7-e^c^	1.17± 0.25 × 10^7^

ALV-B	293 DK-7^a^	1.20 ± 0.30 × 10^7^
ALV-B/Epo	293 or 293T 293T-EpoR^b^	<10^4^ 9.2 ± 2.6 × 10^7^
ALV-B/SCF	MO7-e^c^	8.38 ± 3.60 × 10^6^

^a^Transduction efficiencies of ALV-A and ALV-B pseudotypes were determined on 293 DK-7 cells, a clonal cell line expressing the chimeric TVA/TVB receptor. Vectors used were concentrated 250-fold. The titers of un-concentrated virus were 8.12 ± 4.4 × 10^5 ^(ALV-A) and 2.0 ± 0.9 × 10^5^(ALV-B) respectively.

^b^Pseudotypes preloaded with TVA-Epo or TVB-Epo were used to transduce a 293T-based clonal cell line expressing EpoR. Vectors used were concentrated 250-fold. The titers of un-concentrated virus were 7.27 ± 0.77 × 10^5 ^(ALV-A) and 7.80 ± 0.46 × 10^5 ^(ALV-B) respectively.

^c^Pseudotypes preloaded with TVA-SCF or TVB-SCF were used to transduce c-kit-expressing MO7-e cells. Vectors used were concentrated 250-fold.

^d^Titers presented represent the mean ± SD from three independent experiments using concentrated vector stocks.

### Expression of recombinant TVA and TVB bridge proteins

**Figure 4:**
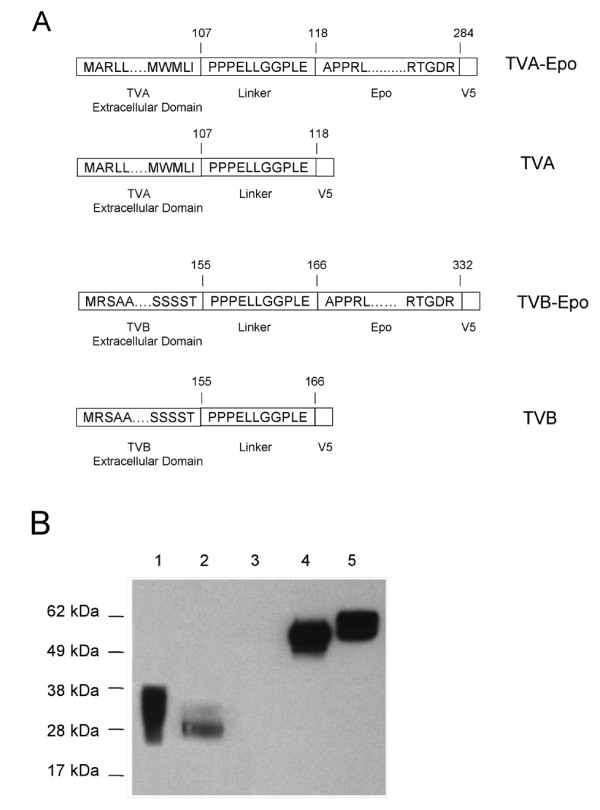
**Analysis of TVA and TVB bridge proteins**. (A) Schematic representation of TVA and TVB fusion proteins. The ends of the unprocessed TVA and TVB extracellular domains including their signal peptide sequences are shown. To generate TVA-Epo and TVB-Epo, the human Epo sequence encoding amino acids 28 to 193 of the mature protein was placed downstream of the linker-encoding sequence. The numbers refer to the ends of the respective protein domains. (B) Western blot analysis of TVA and TVB fusion proteins. Cell supernatants collected from 293T cells stably expressing TVA and TVB proteins were analyzed by Western blotting using monoclonal anti-V5 antibody. Lanes 1, 2, 4, and 5: Supernatants from cells producing TVA, TVB, TVB-Epo and TVA-Epo proteins, respectively. Lane 3: Control 293T cells. The amounts loaded were equalized based on the V5 epitope-specific ELISA assay described in Materials and Methods.

In a next step, bi-functional fusion proteins consisting of a 107 amino acid-long soluble extracelluar domain of the TVA receptor or a 155 amino acid-long soluble extracellular domain of the TVB receptor fused in-frame to the mature form of human Epo and containing a V5 tag sequence were generated (Fig. 4A). A 9-amino acid proline-rich linker sequence [23] derived from the hinge region of the rabbit Fc chain was placed in between the TVA or TVB domains and the Epo sequence (Fig. 4A). The amounts of the fusion proteins released into the cell culture supernatant from stably transfected 293T cell clones were analyzed by an ELISA test using an antibody directed against the V5 epitope present in the fusion proteins. The results from the ELISA assay indicated that the concentrations of the unmodified soluble TVA domain and that of the TVA-Epo fusion protein were in the range of 300 - 800 V5 units/ml while the TVB and TVB-Epo protein levels were in the range of 1,500 - 2,000 V5 units/ml (data not shown).

A Western blot analysis of cell culture supernatants using anti-V5 antibody showed that the sizes of the TVA protein ranged from 28 to 35 kDa, while the sizes of the TVA-Epo protein ranged from 55 to 60-kDa (Fig. 4B). These findings are consistent with the view that posttranslational modifications affected the migration of the TVA proteins in SDS polyacrylamide gels [23]. The TVB protein displayed an apparent molecular weight around 28 kDa which is slightly larger than the expected size. The TVB-Epo protein showed a molecular weight around 55 kDa.

### Cell-specific transduction by lentiviral vectors pseudotyped with ALV-A or ALV-B Env glycoproteins mediated by Epo-EpoR interactions

**Figure 5:**
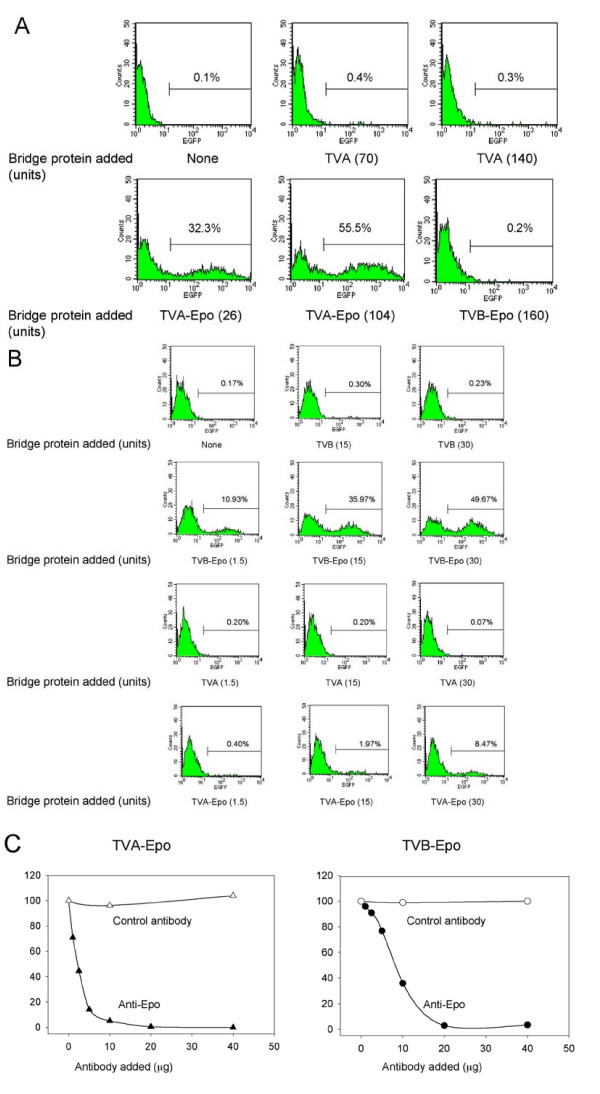
**Transduction of 293T-EpoR cells using ALV-A or ALV-B-pseudotyped lentiviral vectors preloaded with TVA-Epo or TVB-Epo bridge protein**. (A) Specificity of TVA-Epo-mediated transduction. 293T-EpoR cells were transduced with ALV-A-pseudotyped LV-EGFP vectors preloaded with TVA-Epo or TVB-Epo bridge proteins and subjected to FACS analysis three days later. Aliquots of a concentrated vector stock corresponding to 5 × 10^4 ^TU (determined on 293 DK-7 cells) were preincubated with cell culture supernatants containing TVA (upper center panel and upper right panel), TVA-Epo (lower left panel and lower center panel) or TVB-Epo (lower right panel). The amounts of bridge proteins added (expressed as V5 units) are indicated in parentheses. (B) Specificity of TVB-Epo-mediated transduction. 293T-EpoR cells were transduced with ALV-B-pseudotyped vectors preloaded with TVB-Epo and TVA-Epo bridge proteins and subjected to FACS analysis 3 days later. Aliquots of a concentrated vector stock corresponding to 1.2 × 10^4 ^TU (determined on 293 DK-7 cells) were preincubated with cell supernatants containing TVB or TVB-Epo (top two panels), or TVA or TVA-Epo (bottom two rows). The amounts of TVB and TVB-Epo proteins added (expressed as V5 units) are indicated. Representative FACS profiles are shown. (C) Inhibition of TVA-Epo and TVB-Epo-mediated transduction of 293T-EpoR cells by anti-Epo antibody. 293T-EpoR cells were transduced using an EGFP-encoding lentiviral vector pseudotyped with the ALV-A Env (left panel) or the ALV-B Env (right panel). Vectors were exposed to cell supernatants containing TVA-Epo in the presence of monoclonal anti-Epo antibody or monoclonal anti-Flag antibody (as a control) on ice for 30 min. Transduced cells were subjected to FACS analysis three days later. The percentages of EGFP-positive cells are indicated. Representative data from two independent assays are shown.

To test the capacity of TVA-Epo and TVB-Epo fusion proteins to act as a bridge to target lentiviral vectors pseudotyped with the ALV-A and ALV-B Env proteins to cells expressing EpoR, 293T-EpoR cells were transduced with LV-EGFP particles that had been preloaded with the TVA-Epo or TVB-Epo fusion proteins. As shown in Fig. 5A (bottom left and middle panels), LV-EGFP vectors pseudotyped with the ALV-A Env glycoprotein after pre-incubation with the TVA-Epo fusion protein transduced 293T-EpoR cells efficiently while vectors that had been pre-incubated using unmodified TVA (top middle and right panels) or TVB-Epo (bottom right panel) did not.

Similar assays were performed with ALV-B pseudotypes pretreated with TVB and TVA fusion proteins or unmodified TVB or TVA. As indicated in Fig. 5B, 293T-EpoR cells were transduced efficiently using vectors pre-incubated with TVB-Epo proteins (Fig. 5B, second row), but not with vectors pre-incubated with unmodified TVB or TVA proteins (Fig. 5B, rows one and three). Interestingly, however, unlike the ALV-A pseudotypes which did not transduce 293T-EpoR cells efficiently after pre-incubation with TVB-Epo proteins, low levels of transduction were observed when the cells were transduced using ALV-B-pseudotyped vectors bearing TVA-Epo proteins (Fig. 5B, row four), possibly indicating a weak interaction between the ALV-B Env glycoprotein and TVA-Epo. Transduction of 293T cells using ALV-B pseudotypes containing TVB-Epo was below the level of detection (data not shown).

The titers of ALV-A and ALV-B pseudotypes preloaded with TVA-Epo or TVB-Epo bridge proteins produced under optimized conditions after a 250-fold concentration by ultracentrifugation were 8.60 ± 0.70 × 10^7 ^TU/ml and 9.2 ± 2.6 × 10^7 ^TU/ml, respectively (Table 1). These titers compare favorably with those obtained using 293 DK-7 cells expressing the chimeric TVA-TVB receptor which were 1.57 ± 0.47 × 10^8 ^TU/ml for pseudotypes bearing the ALV-A Env and 1.20 ± 0.30 × 10^7 ^TU/ml for pseudotypes bearing the ALV-B Env (Table 1), indicating that bridge-mediated transduction was very efficient.

To demonstrate that Epo/EpoR-mediated transduction was specific, a monoclonal anti-Epo antibody was included during the preloading step of the vector pseudotypes with the bridge proteins. As shown in Fig. 5C, the transduction of EpoR-expressing cells was inhibited in a dose-dependent manner when increasing amounts of the anti-Epo antibody were included, while a control monoclonal antibody (anti-Flag) had no effect.

### Incorporation of bridge proteins during lentiviral vector production

**Figure 6:**
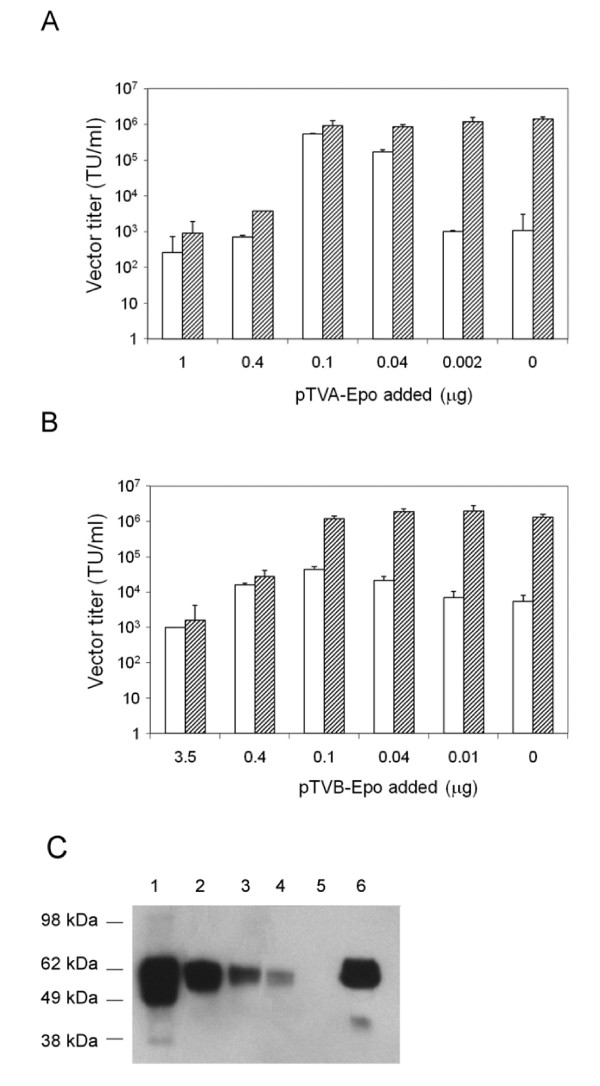
**Transduction of 293T-EpoR cells using lentiviral vector pseudotypes preloaded with TVA-Epo or TVB-Epo during vector production**. (A) Open bars: ALV-A Env-pseudotyped lentiviral vectors prepared in 6-well plates using different amounts of the pTVA-Epo plasmid ranging from 0 to 1.0 μg. Striped bars: *In vivo*-preloaded vectors were subjected to a subsequent preloading step *in vitro *using the TVA-Epo protein (40 V5 units of TVA-Epo per 50 μl of virus stock). (B) Open bars: ALV-B Env-pseudotyped lentiviral vectors prepared in 6-well plates using different amounts of pTVB-Epo ranging from 0 to 3.5 μg. Striped bars: *In vivo*-preloaded vectors were subjected to a subsequent preloading step *in vitro *using the TVB-Epo protein (14 V5 units of TVB-Epo per 50 μl of virus stock). The titers shown in panels A and B represent the mean ± SD from three independent experiments. (C) Western blot analysis of ALV-A-pseudotyped lentiviral vectors prepared by co-transfection with different amounts of pTVA-Epo. Lanes 1-5: Two-μl aliquots of vector stocks prepared by co-transfection using 0.5 μg (lane 1), 0.1 μg (lane 2), 0.04 μg (lane 3), 0.02 μg (lane 4) and 0 μg (lane 5) of the pTVA-Epo plasmid DNA and concentrated ~35-fold by ultracentrifugation were analyzed. Lane 6: Unconcentrated vectors prepared using 0.4 μg of the pTVA-Epo plasmid DNA. The molecular weights of the protein markers used are indicated.

Boerger et al. have shown that Moloney murine leukemia virus-based vectors pseudotyped with the ALV-B Env protein and decorated with TVB fusion proteins could be produced directly from vector packaging cells [24]. This is an attractive feature in that the *in vitro *preloading step can be bypassed. To test whether lentiviral vectors bearing ALV-A or ALV-B Env glycoproteins could be preloaded with TVA-Epo or TVB-Epo fusion proteins during vector production, vectors were prepared by co-transfecting 293T cells with four different plasmids including an EGFP-expressing lentiviral vector plasmid, a packaging plasmid, an ALV-A or ALV-B Env plasmid, and the pTVA-Epo or pTVB-Epo plasmids. As shown in Fig. 6, vector titers were critically depended on the amounts of the pTVA-Epo or pTVB-Epo plasmids added during vector production. The highest titers reached for TVA-Epo-containing particles were 5.3 ± 0.3 × 10^5 ^TU/ml (Fig. 6A, open bars). Subjecting these *in vivo*-preloaded vectors to a subsequent preloading step *in vitro *using the TVA-Epo protein had an additive effect on the titers depending on the amount of the TVA-Epo plasmid added during the transfection step (Fig. 6A, striped panels). The highest titers obtained in this way were 1.2 ± 0.4 × 10^6 ^TU/ml.

Similar experiments were conducted by including pTVB-Epo during preparation of ALV-B pseudotypes (Fig. 6B). The highest titers obtained were 4.4 ± 0.9 × 10^4 ^TU/ml (Fig. 6B, open bars). However, the titers increased substantially following exposure of such pseudotypes to the TVB-Epo protein *in vitro *reaching titers around 2.0 ± 0.8 × 10^6 ^TU/ml (Fig. 6B, striped bars). It is also evident from Fig. 6A and 6B that the relative amounts of the bridge proteins present during vector loading post-production or during production critically affect vector titers.

In order to confirm that bridge proteins were specifically bound to vector particles, ALV-A pseudotypes were concentrated by ultracentrifugation. Western blot analyses using anti-V5 antibody revealed the presence of TVA-Epo on vector particles after concentration. Furthermore, the relative levels of the bridge proteins detected in concentrated vector particles correlated well with the amounts of pTVA-Epo used during vector production (Fig. 6C).

### Cell-specific transduction by lentiviral vectors pseudotyped with ALV-A or ALV-B glycoproteins mediated by SCF-c-kit interactions

In order to test if bridge protein-mediated transduction by lentiviral vectors can be applied to other ligands and cell lines, bi-functional proteins containing the soluble extracellular domain of TVA or TVB fused to the soluble portion of human SCF were generated and tested in MO7-e cells that express c-kit. As shown in Table 1, the titers of concentrated ALV-A (ALV-B) pseudotypes preloaded with TVA-SCF (or TVB-SCF) in MO7-e cells ranged from 10^6 ^to 10^7 ^TU/ml.

## Discussion

Various strategies have been explored to target lentiviral vector delivery to specific cells *in vitro *and *in vivo *including approaches involving engineered versions of the Sindbis virus E2 glycoprotein bearing either a *Staphylococcus aureus *protein A domain [4-13], or single chain antibody fragments [15]. In a related targeting strategy, the cell recognition function and the fusion function mediated by the Sindbis virus E2 glycoprotein are carried out by separate proteins that are anchored in the vector's membrane [17-19]. A drawback with these approaches is that they involve glycoproteins derived from laboratory-adapted strains of Sindbis virus necessitating the introduction of mutations to abrogate high efficiency binding to cell surface receptors such as heparan sulfate. Unfortunately, background transduction levels in the absence of the ligand or with cells lacking the corresponding receptors were substantial due to the leakiness of the mutations that were introduced to abolish cell binding [6].

Alternative approaches for lentiviral vector targeting are emerging. For example, in a recent report Funke et al. [35] have shown that the measles virus hemagglutinin (H) and fusion protein (F) are capable of pseudotyping of HIV-1 vectors. Moreover, engineered H proteins displaying cell-specific ligands or single chain antibodies resulted in specific transduction of target cells. While the background transduction levels in the absence of the ligand were low, the concentration of such pseudotypes by centrifugation has been challenging.

In this study, we investigated the efficiency and selectivity of a cell targeting approach involving lentiviral vectors pseudotyped with the Sindbis virus strain TR339 glycoproteins [30] plus a separate cell binding domain. The Sindbis virus TR339 strain was originally derived from the AR339 strain which represents the prototype alphavirus and whose sequence differs markedly from that of the common Sindbis virus laboratory strains that have emerged after selection for efficient growth in cell culture [36]. Such laboratory strains bear mutations that facilitiate binding of the virus to heparan sulfate [30], a property that is clearly not desirable in the context of targeting stategies. Our data indicate that the titers of SCF-containing vectors bearing the Sindbis virus TR339 glycoproteins were up to 3.2 ± 0.04 × 10^5 ^TU/ml on c-kit-expressing 293 cells, while the titers of lentivirus particles lacking SCF were some 80 fold lower (Fig. 1C). These background transduction levels are comparable to those reported previously by Morizono et al. [6] using multiply mutated versions of a Sindbis virus laboratory strain-derived E2 protein. However, it is likely that the unmodified E2 protein of the TR339 strain retains considerably more of the non-attachment activity of this protein than the highly modified version used by Morizono et al. Therefore, it is possible that use of the wild type E2 may improve the efficiency of pseudotype particle production. However, a direct comparison of infectivity per particle of the different vectors will be required to answer this question.

We also explored a targeting approach involving vector particles containing a membrane-proximal fusion domain (VSV-GS) derived from the VSV G glycoprotein [29] plus a separate cell binding domain. The results reported earlier by Jeetendra et al. [29] showed that the membrane-proximal 42 amino acids (residues 421 to 461) of the VSV-G protein ectodomain together with the TM region and the cytoplasmic tail were able to potentiate the membrane fusion activity of heterologous viral fusion proteins when the two proteins were co-expressed. We investigated the efficiency of the VSV-GS domain to target SCF-displaying lentiviral vector particles to c-kit expressing cells. The results presented in Figs. 2B and 2C show that specific transduction of c-kit-expressing 293 and MO7-e cells was achieved and that transduction efficiencies using SCF-bearing vectors were 40 fold higher (1.4 ± 0.2 × 10^4^) compared to vectors lacking SCF (3.5 ± 1.5 × 10^2^). Overall, the titers obtained with VSV-GS-containing particles were low, possibly because VSV-GS mostly promoted hemifusion events [29]. It was evident that MO7-e cells were transduced less efficiently than 293-c-kit cells although c-kit levels were higher in MO7-e cells as judged by FACS (data not shown). This may be due to the fact that the transduction conditions used for MO7-e cells were not optimal. This view is enforced by the observation that VSV-G pseudotypes on MO7-e cells did relatively poorly compared to 293-c-kit cells (data not shown).

It was interesting to note that lentiviral vectors bearing SCF, but lacking the VSV-GS fusion domain, were capable of transducing 293-c-kit cells but not 293T cells (Fig. 2B). This may indicate that SCF provided some cryptic fusion function. Furthermore, the transduction experiments involving 293T cells presented in Fig. 1D revealed that transduction efficiencies of vector particles containing SCF increased in a dose-dependent manner. This may have resulted from the binding of vector particles to 293T cells through nonspecific SB-Env interactions and augmented fusion triggered by SCF.

Young and collaborators have shown that alpharetroviral and gammaretroviral vectors carrying unmodified ALV envelope glycoproteins and bearing specific ligand proteins or single chain antibody as a bridge were capable of targeting specific cells *in vitro *[23-27]. An attractive feature of this bridge targeting system is that the background transduction levels were low. These findings promoted us to investigate the capacity of lentiviral vectors bearing the ALV-A and ALV-B Env glycoproteins to target specific cells *in vitro *using soluble TVA and TVB bridge proteins bearing cell-specific ligands. Fig. 3 shows that HIV-1-based vectors were efficiently pseudotyped using both the ALV-A and ALV-B Env glycoproteins as judged from transduction experiments involving 293-DK7 cells that express a hybrid ALV-A/B receptor [34]. The titers obtained were 1.57 ± 0.47 × 10^8 ^TU/ml for pseudotypes bearing the ALV-A Env protein after a 250-fold concentration and 1.20 ± 0.30 × 10^7 ^TU/ml for ALV-B pseudotypes (Table 1). The ALV-A pseudotype titers obtained compare favorably to those reported previously by Lewis et al. 2001 [37]. It was interesting to note, however, that the titers reported by Lewis et al. involving an unmodified version of the ALV-A Env protein were considerably lower, less than 10^4 ^TU/ml, while ALV-A glycoproteins bearing a truncated cytoplasmic tail or a chimeric cytoplasmic tail were some 5-fold higher. Our work involved unmodified ALV-A Env and ALV-B Env proteins bearing intact cytoplasmic tails. Yet the titers were consistently well above 10^5 ^TU/ml for both ALV-A and ALV-B pseudotypes (Table 1). The reason for this discrepancy is not clear; it may be due to differences in the TVA-expressing 293 cell lines that were used for vector titration.

Boerger et al. [24] have previously shown that retroviral vectors preloaded with TVB-EGF bridge proteins were relatively thermostable and could be generated directly from vector-producing cells. Our findings show that the same approach is feasible as far as TVB-Epo-containing lentiviral vector particles are concerned (Fig. 6B). Interestingly, the same approach was applicable to TVA-Epo-containing lentiviral vectors as well (Fig. 6A). This was possibly facilitated by metastable fusion-incompetent ALV-A Env/TVA-Epo complexes that were unable to complete the fusion reaction during vector production at neutral pH [38,39].

An attractive feature of the bridge strategy involving ALV-A Env pseudotyped vectors is that background levels were low (Fig. 5A). However, the same kind of tight specificity was not seen with ALV-B-pseudotyped lentiviral vector particles. Such particles displayed residual transduction in the presence of the heterologous TVA-Epo bridge protein but not in the presence of unmodified TVA alone. The reason for this difference in the binding specificities is not clear at present.

## Conclusions

In summary, the bridge approach provides a promising strategy for cell-specific targeting of lentiviral vectors. The most promising results were obtained using ALV-A Env-pseudotyped vectors decorated with soluble TVA receptors bearing cell-specific ligands. A chief advantage of this strategy compared to other lentiviral targeting strategies includes lower background transduction. What remains to be determined in the future are the relative transduction efficiencies and specificities *in vivo *of the pseudotypes made with the different approaches.

## Methods

### Plasmids

Plasmid pcDNA-SB-Env encoding the Sindbis virus strain TR339 E3, E2, 6K and E1 proteins (Fig. 1A) [30,32] was generated as follows: A synthetic DNA fragment (160 bp) encoding the first 45 amino acids of the E3 protein with an *Apa*I site at the 5'-end and a *Stu*I site at the 3' end was used to replace the *Apa*I-*Stu*I fragment present in the Sindbis replicon glycoprotein helper pINT [30]. From the resulting plasmid, a 3.3-kb *Hin*dIII-*Xho*I DNA fragment was subcloned into pcDNA3.1/Zeo(+) (Invitrogen, Carlsbad, CA) to generate pcDNA-SB-Env. An expression plasmid encoding a membrane-bound form of human SCF was generated by subcloning a *Hin*dIII-*Bam*HI fragment derived from the pBluescriptSK-hMGF plasmid (a gift from Paul Schwarzenberger, LSU Health Sciences Center, New Orleans, LA) into the pUB6/V5-HisB expression plasmid (Invitrogen), resulting in pUB6-HuMGF. A synthetic DNA fragment encoding the membrane-proximal stem region of the VSV-G ectodomain (G stem), transmembrane and cytoplasmic domains (amino acids 395 to 511) [28,29] was subcloned into the expression vector pUB6/V5-HisB to generate pUB6-VSV-GS. Plasmid pCB6 EnvA encoding the ALV-A Env glycoprotein [40] was obtained from Galen Fisher (National Cancer Institute, Bethesda, MD). Plasmid pAB7 encoding the ALV-B Env glycoprotein [24] was kindly provided by John Young (University of Wisconsin, Madison, WI). A DNA fragment encoding the extracellular domain of TVA (amino acids 1-107) linked in-frame to a proline rich sequence (PPPELLGGP) was PCR-amplified using pCB6 Tva950 [41] (a gift from Galen Fisher) as a template and primers TVA-F (5'-GTTCTAGCTAGCGCTGTGCGCGGTACCGATATG-3') and TVA-Linker-R (5'-GATTCCCTCGAGCGGTCCCCCCAGGAGTTCAGGGGGTGG-3'). The PCR product was digested with *Nhe*I and *Xho*I and cloned into pcDNA6/V5-His (Invitrogen) cut with *Nhe*I and *Xho*I, resulting in pTVA-V5 which encodes the TVA-PPPELLGGP protein fused in-frame to a V5 epitope sequence. Similarly, pTVB-V5 was generated by cloning a PCR fragment encoding the extracellular domain of TVB (amino acids 1 to 155) fused in-frame to the PPPELLGGP sequence into pcDNA6/V5-His. To do this, pBK7.62 (a gift from John Young) [42] was used as a template for PCR. The primers used were TVB-F (5'-GTTCTAGCTAGCGAGATGCGCTCAGCTGCGCTCC-3') and TVB-Linker-R (5'-GATTCCCTCGAGCGGTCCCCCCAGGAGTTCAGGGGGTGGAGTGGAGGAGCTGGAGGAGAT-3'). To construct plasmids pTVA-Epo and pTVB-Epo encoding TVA-Epo and TVB-Epo fusion proteins, respectively, a 0.5 kb fragment encoding amino acids 1 to 166 of the mature erythropoietin protein [43] was generated by PCR using plasmid R576 as a template (A gift from Lorraine Albritton, University of Tennessee Health Science Center, Memphis, TN) and a forward primer (5' CCCTCGAGGCCCCACCACGCCTCATC-3') and a reverse primer (5'-GAAGGGCCCTCTGTCCCCTGTCCTGCA-3'). This fragment was digested with *Xho*I and *Apa*I and subcloned into pTVA-V5 and pTVB-V5, respectively. A similar strategy was used to generate pTVA-SCF and pTVB-SCF in which sequences encoding the soluble portion of human SCF (amino acids 1 to 165) [44] were fused in-frame to TVA or TVB sequences. DNA sequences encoding human SCF were generated by PCR using pBluescriptSK-hMGF as a template and forward primer 5'-GAGCTCGAGGAAGGGATCTGCAGGAATCGT-3' and reverse primer 5'-TTAGGGCCCGGCTGCAACAGGGGGTAACAT-3'. Plasmid pcDNA-EpoR encoding a full length human Epo receptor was generated by sucloning an EpoR cDNA (pCMV6-XL4-EpoR, Origen, Rockville, MD) into pcDNA3.1/Zeo(+). Plasmid pCMV6-XL5-c-kit-IRES-Neo, encoding c-kit was generated by inserting IRES-Neo sequences which were derived from plasmid pMGIN [45] downstream of the c-kit cDNA sequence present in pCMV6-XL5 c-kit (Origen). The pNL-EGFP/UbC/WPREΔU3 [46], pNL-EGFP/CMV/WPREΔU3 [47], pNL-LacZco/CMV/WPREΔU3 [48] and pCD/NL-BH* ΔΔΔ [49] plasmids were described before.

### Cell lines

Human embryonic kidney 293 (CRL-1573) and 293T cells (CRL-11268) were obtained from the American Type Culture Collection (ATCC, Manassas, VA). Cells were maintained in DMEM containing 10% heat inactivated FBS, 2.5 mM L-glutamine, 100 u/ml penicillin and 100 μg/ml streptomycin (all from Invitrogen-GIBCO). 293/DK-7 cells were generated by stable transfection with plasmid pDK7 expressing a chimeric TVA-TVB receptor [34] (a gift from John Young). Cell clones were maintained in medium containing 500 μg/ml of G418 (Invitrogen). 293T-EpoR cells were established by stable transfection using the pcDNA-EpoR plasmid and cell clones were maintained in medium containing 1 mg/ml of Zeocin (Invitrogen). 293-c-kit cells were established by stable transfection using the pCMV6-XL5-c-kit-IRES-Neo plasmid and cell clones were maintained in medium containing 500 μg/ml of G418. 293T cells stably expressing TVA, TVA-Epo, TVA-SCF, TVB, TVB-Epo, or TVB-SCF were generated by transfection with the plasmids described above and selection using media containing 10 μg/ml of blasticidin (Invitrogen). MO7-e cells (obtained from Paul Schwarzenberger) were maintained in RPMI 1640 medium (Sigma-Aldrich, St Louis, MO) containing 20% heat inactivated FBS (Invitrogen-GIBCO), 2.5 mM L-glutamine, 100 u/ml penicillin, 100 μg/ml streptomycin (all from Invitrogen-GIBCO) and 10 ng/ml of GM-CSF (Sigma).

### Lentiviral vector production

Lentiviral vector stocks were prepared as described before [50]. To prepare small-scale stocks using 6-well plates, 5 μg aliquots of the pNL-EGFP/UbC/WPREΔU3, pNL-EGFP/CMV/WPREΔU3 or pNL-LacZco/CMV/WPREΔU3 plasmid DNAs, plus 3.5 μg of the pCD/NL-BH* ΔΔΔ helper plasmid and 3.5 μg of the pCB6 EnvA or pAB7 Env plasmids were mixed. For vectors bearing Sindbis virus strain TR339 glycoproteins and SCF, 5 μg of the pNL-EGFP/UbC/WPREΔU3 vector plasmid, 3.5 μg of the pCD/NL-BH* ΔΔΔ helper plasmid, 0.25 μg of the SB-Env plasmid and varying amounts of the pUB-HuMGF plasmid were used. To prepare large-scale vector stocks using 150 mm dishes, 21 μg of the pNL-EGFP/UbC/WPREΔU3 or pNL-LacZco/CMV/WPREΔU3 plasmid DNAs plus 14 μg of the pCD/NL-BH* ΔΔΔ helper plasmid and 21 μg of the pCB6 EnvA or pAB7 Env plasmid DNAs were mixed. For concentrated vectors bearing the VSV-GS fusion protein and SCF, 21 μg of the pNL-EGFP/UbC/WPREΔU3 or pNL-LacZco/CMV/WPREΔU3 plasmid DNAs plus 14 μg of the pCD/NL-BH* ΔΔΔ helper plasmid, 21 μg of pUB6-VSV-GS and 21 μg of pUB6-HuMGF were mixed. For concentrated vectors bearing the Sindbis virus strain TR339 glycoproteins and SCF, 21 μg of the pNL-EGFP/UbC/WPREΔU3 plasmid DNAs plus 14 μg of the pCD/NL-BH* ΔΔΔ helper plasmid, 2.1 μg of pcDNA-SB-Env and varying amount of pUB6-HuMGF were mixed. Vector stocks were concentrated by ultracentrifugation as described before [50]. p24 antigen ELISA tests were carried out as described [50].

### Transduction of cells using vectors bearing fusion domains

Transductions were performed as described before [51] with the following modifications: 5 × 10^4 ^293T or 293-c-kit cells were plated in 12 well plates. Twenty hours later cells were transduced using lentiviral vectors bearing the Sindbis virus strain TR339 glycoproteins or VSV-GS (with or without human SCF) in the presence of polybrene (8 μg/ml). Cells were subjected to FACS analysis three days later. For transduction of MO7-e cells, 2 × 10^5 ^cells were mixed with vector aliquots in 0.2 ml medium containing polybrene (8 μg/ml) in 12 × 75 mm tubes, followed by spinoculation at 1,000 rpm at 25°C for 180 min and incubation at 37°C for 15 hours. Cells were re-fed with 0.8 ml of medium and analyzed by FACS 72 hours later.

### Transduction of cells using bridge strategy

5 × 10^4 ^293T-EpoR cells were plated in 12-well plates. Twenty hours later, the plates were placed on ice for 1 hour. Lentiviral vectors pseudotyped with the ALV-A or ALV-B Env proteins were mixed with different amounts of the bi-functional bridge proteins in the presence of 40 mM HEPES buffer (pH 7.0) and kept on ice for 30 min. Preloaded lentiviral vectors were added to pre-chilled cells in 0.3 ml of ice-cold medium containing polybrene (8 μg/ml). Cells were kept on ice for 30 min before transferring them to a 37°C incubator. Alternatively, 0.4 ml of ice-cold medium containing different amounts of the bridge protein were added following preincubation of the cells on ice for 1 hour. The cells were kept on ice for another hour. Cells were then washed with ice-cold medium and transduced with lentiviral vectors pseudotyped with ALV-A (or ALV-B). Cells were subjected to FACS analysis 72 hours post-transduction. For transduction of MO7-e cells, 2 × 10^5 ^cells were chilled on ice for 1 hour and mixed with preloaded vector aliquots in 0.2 ml ice-cold medium containing polybrene (8 μg/ml) in 12 × 75 mm tubes. The cells were transduced by spinoculation at 1,000 rpm at 25°C for 180 min and incubated at 37°C for 15 hours. Cells were re-fed with 0.8 ml medium and analyzed by FACS 72 hours later. Competition experiments were performed by adding anti-human Epo antibody or anti-human SCF antibody (R & D Systems Inc. Minneapolis, MN) as indicated in the figure legends. Cells were washed and fresh medium was added. Cells were subjected to FACS analysis as described above.

### Quantitation of TVA and TVB fusion proteins by ELISA

To determine the relative amounts of the bridge proteins produced, an ELISA assay based on the V5 epitope present in all TVA and TVB proteins was established. To determine bridge protein levels, 5 × 10^6 ^293T cells expressing TVA, TVA-Epo, TVA-SCF, or TVB, TVB-Epo or TVB-SCF were seeded in T75 flasks with 15 ml medium containing blasticidin (10 μg/ml). Cell supernatants were recovered three days later and centrifuged at 1500 rpm for 5 min to remove cell debris. Aliquots of the supernatants were stored at -80°C. The relative amounts of the bi-functional fusion proteins released by the cells were determined using an ELISA test. This test was performed as follows: 50 μl aliquots of serial dilutions of cell supernatants or the purified V5 peptide (Sigma) in culture medium were used to coat 96-well plates (Nunc Maxisorp, Nalge Nunc International, Rochester, NY) at 25°C overnight. The plates were washed with phosphate-buffered saline (PBS) and incubated with 400 μl blocking solution (PBS containing 0.05% Tween 20 and 5% nonfat milk, PBSTM) at 25°C for 2 hours, followed by incubation with 50 μl of monoclonal anti V5 antibody (Invitrogen, 1: 2,500 dilution in PBSTM) for 2 hours. After extensive washing with PBST, 50 μl of goat anti-mouse IgG conjugated with HRP (BioRad Hercules, CA, 1: 5,000 dilution in PBSTM) was added to each well and the plates were incubated at 25°C for 1 hour and then washed. The plates were processed for staining using 3,3',5,5'-tetramethylbenzidine (TMB, Sigma-Aldrich) as a substrate. The concentrations of the bi-functional proteins were determined using the purified V5 peptide (Sigma) as a standard. The concentrations are referred to as V5 equivalent units/ml.

### Analysis of TVA and TVB fusion proteins by Western blotting

For Western blots [52] 2 to 20 μl aliquots of cell culture supernatants were collected from transfected 293T cells. Alternatively, lentiviral vector stocks (unconcentrated or concentrated by ultracentrifugation) were analyzed. Samples were treated with 50 mM Tris-HCl (pH 7.0) buffer containing 1% SDS, 5% β-mercaptoethanol, 12.5% glycerol and 0.01% bromophenol blue at 95°C for 5 min and subjected to SDS-PAGE using 4-12% NuPAGE Bis-Tris gels in MES buffer (Invitrogen) and blotted onto a PVDF membrane (Millipore, Bedford, MA). The membrane was blocked with 5% nonfat milk in TBST solution (Sigma-Aldrich), and incubated with monoclonal anti V5 antibody (Invitrogen, 1: 2,500) followed by HRP-conjugated goat anti-mouse IgG (BioRad 1:5,000). Blots were treated using the SuperSignal West Pico Chemiluminescent substrate reagent (Pierce Biotechnology Inc., Rockford, IL) and exposed to X-ray films.

## Competing interests

The authors declare that they have no competing interests.

## Authors' contributions

XYZ designed and constructed the recombinant plasmids and carried out the transduction experiments, FACS analyses and Western blots. AB participated in the construction and analysis of the recombinant plasmids. RK and MM helped with vector production and titration. JR, XYZ and WBK conceived of the study, and participated in its design and coordination and helped to draft the manuscript. JR and XYZ wrote the manuscript. All authors read and approved the final manuscript.
